# Opioid prescription patterns in the province of Las Palmas, Canary Islands, Spain (2016–2020): differences between urban and rural areas

**DOI:** 10.3389/fphar.2023.1184457

**Published:** 2023-07-18

**Authors:** Alexis Oliva, Patricia González de Chavez, Sandra Dévora, Susana Abdala

**Affiliations:** ^1^ Departamento de Ingeniería Química y Tecnología Farmacéutica, Facultad de Farmacia, Universidad de La Laguna, Tenerife, Spain; ^2^ Departamento de Medicina Física y Farmacología, Facultad de Farmacia, Universidad de La Laguna, Tenerife, Spain

**Keywords:** opioid prescription, wholesaler, defined daily dose, socio-economic status, strong opioids, rural, Canary islands, Spain

## Abstract

**Introduction:** The use of opioids has increased markedly in the past decades in European countries, especially for treatment of non-cancer pain including painful chronic musculoskeletal conditions. However, there are some notable differences in the relative levels of use between geographical areas and some distinct, context-specific patterns of weak and strong opioid use. The aim of this work is to describe real world trends in dosage forms and population exposure in the prescription opioid use on isolated geographically area: The Canary Islands of Gran Canaria, Lanzarote and Fuerteventura, Spain. For this, several factors such as living in a rural or urban area, population over 65 years of age, population density or socioeconomic status were analyzed.

**Methods:** Data were extracted from the wholesalers who supply the community pharmacies at the population level. Prescription opioid use was measured as defined daily doses (DDD) per 1,000 inhabitants per day. A model based on covariance analysis with two nested fixed factors and one co-variable was used for contrast analysis at different level.

**Results:** The overall DDD per 1000 inhabitants per day and year variation rate in Spain was very similar to that obtained for Gran Canaria and Fuerteventura (0.967 vs. 1.006), although the levels of dispensation were different (14.75 *versus* 18.24 for Gran Canaria and 12.7 for Fuerteventura, respectively). Lanzarote is completely different in all issues, where the opioid consumption rate remained stable during the study period, but with a decreasing tendency. The dispensation level of strong opioids varied between islands, from 56.41% for Fuerteventura vs. 17.61% for Gran Canaria, although these values remained stable. Tramadol with acetaminophen and Tramadol in monotherapy were the most consumed forms of the weak opioids, whereas Buprenorphine was the most used strong opioid followed by Fentanyl, although demand for it varied between islands, the transdermal formulations were the most frequent pharmaceutical preparation.

**Conclusion:** The differences in prescription opioid use are most likely explained by the opioid prescribing practices in each island, whereas factors such urbanicity level, population age, population density and status socioeconomic does not help to explain the differences in prescription opioid use across rural and urban areas.

## 1 Introduction

Chronic pain including the musculoskeletal pain are an important public health problem, responsible for disabilities, distress, and quality of life impairment (de Sola et al., 2016; Gorasso et al., 2023). Among the musculoskeletal (MSK) conditions that cause the most disability in terms of years lived with disability are low back pain, neck pain, osteoarthritis and rheumatoid arthritis. Furthermore, according to the 2019 Global Burden of Disease (GBD) study, low back pain and osteoarthritis are the largest contributors to years of productive life lost in the workforce compared to other noncommunicable diseases (GBD, 2020). The physiopathology of chronic pain has been recognized to involve complex interactions between physical, psychological and social factors, and that its appropriate management requires both pharmacological and non-pharmacological treatment (Broekmans et al., 2010). Although opioids were originally formulated for acute and cancer pain, their use has expanded to the treatment of severe acute pain and moderate to severe chronic pain that does not respond to other medications (British Pain Society, 2012).

The prevalence and factors associated with the use of opioids among patients with chronic non-cancer pain (CNCP) was analyzed by De Sola et al. (2020) using a meta-analysis. In this study, factors such as being male, young age, polymedicated patients, smokers and payment type, were associated with higher prevalence rates. However, non-white and Asian patients, and those treated by a physician trained in complementary medicine were less likely to use opioids.

However, other studies suggest that women are more likely to be prescribed opioids in CNCP. Perelló-Bratescu et al. (2022) analyzed the trends in the prescription of strong opioids for this pathology in primary care in Catalonia, Spain, using a descriptive, longitudinal, retrospective population-based study from 2013 to 2017. This study showed that there was a sustained increase in the prescription of strong opioids at high doses, and in mainly elderly patients, predominantly low-income women. Birke et al. (2016) analyzed the prescription of opioids in the Denmark population from 2010 to 2013. In this study, women with CNCP, especially >65 years, became more frequent users of opioids and used higher doses than men. Serdarevic et al. (2017) suggest that older women living alone have higher rates of prescription opioid use.

Other studies have reported that factors such as urbanicity level (living in a rural or urban area) demographic variables (sex and gender), population over 65 years of age, employment rate, socio-economic status, etc. may also explain the possible differences in prescription opioid use. For example, Davies et al. (2019) examined the patterns in opioid prescribing for CNCP in Wales, using a retrospective cross-sectional study. This study showed the associations between higher rates of opioid prescribing and lower socio-economic conditions. Areas of greatest deprivation had more than twice the rate of opioid prescription than the least deprived areas. Similar results were reported in another countries (Davies et al., 2019; Pear et al., 2019; Böckerman et al., 2021a; Nowakowska et al., 2021). Serra-Pujadas et al. (2021) analyzed the socioeconomic and gender inequalities in opioid use in the different geographical areas of Catalonia, Spain. The results show that socio-economic status has a major impact on the use of opioids, with the most deprived areas having the highest levels of opioid use. In addition, and particularly in these areas, women have much higher rates of use of weak opioids than men. Bedene et al. (2019) analyzed the risk factors associated with opioid use in the Netherlands from 2013 to 2017. In this study, female sex, older age, lower socioeconomic status, smoking, and obesity were associated with an increased risk of opioid prescription. Additionally, they observed that poor self-perceived health, depressive symptoms and loneliness, lower household income, and being divorced or widowed were associated with opioid prescription.

Keyes et al. (2014) evaluated the rural-urban differences in prescription opioid use in the US using specific factors. According to these authors, social norms, cultural traditions, attitudes, availability and policies might explain the broad differences in the prevalence of opioid use across rural and urban areas. The rural areas were found to have higher rates of opioid prescription, higher-rates of high-dose opioid prescription or fewer resources for inpatient and outpatient opioid treatment, etc., in comparison to urban areas (Keyes et al., 2014; Sears et al., 2020; Shoff et al., 2021).

Böckerman et al. (2021b) investigated the effect of prescription opioid use on labor market participation, employment and unemployment, using nationwide data from Finland. They found that increased opioid use led to worse labor market outcomes in the long run.

Although prescription opioid use has been studied in various countries in the last two decades (García del Pozo et al., 2008; Hamunen et al., 2009; Hastie et al., 2014; Alexander et al., 2018; Hider-Mlynarz et al., 2018; Bosetti et al., 2019; Chenaf et al., 2019; Zhu et al., 2019), few studies in Europe have focused on local zones such as region, island, municipalities or health areas to establish a prescription pattern of opioid use. For example, Mordecai et al. (2018) examined patterns of regional variation of opioid prescription in primary care in England during the period 2010–2014. Ashaye et al. (2018) analyzed the regional differences in opioid prescription between two UK geographical areas -London and the Midlands. However, the differences between areas were not significant. Curtis et al. (2019) used a retrospective database study to examine opioid prescribing trends and geographical variation in England between 1998 and 2018. Higher rates of high-dose prescribing were associated with practice list size, rural location and deprivation. However, there was wide geographical variation across England. Serra-Pujadas et al. (2021) conducted a similar study in different geographical areas of Catalonia, Spain. The most deprived areas had the highest levels of opioid use. Oliva et al. (2022) recently evaluated the prescription opioid use on the island of La Gomera in the Canary Islands, a mainly rural area, during the period 2016–2020 at various levels. The differences in prescription opioid use are most likely explained by prescriber characteristics, whereas population age, socioeconomic status, or living in a rural/urban area are not decisive determinants.

The aim of this work is to analyze the use of prescription opioids in the three islands that make up the province of Las Palmas, Canary Islands, Spain (Gran Canaria, Lanzarote and Fuerteventura) as an example of an isolated geographical area with an urban and rural population. To do this, a drug utilization study based on the Anatomical Therapeutic Chemical Classification (ATC) and defined daily dose (DDD) methodology was used (WHO, 2018). In this context, a model, based on covariance analysis (ANCOVA) with two nested fixed factors and one co-variable, was used for the contrast analysis. For this purpose, the raw data obtained from the wholesalers that supply the community pharmacies at the population level were used as a database, which is a novel approach.

## 2 Materials

### 2.1 Data

The study of prescription opioid use at the outpatient level was conducted using the ATC/DDD methodology, which is internationally accepted for measuring drug utilization within and across populations. The different opioid types were identified in the database by their ATC-code. Opioids indicated for analgesia all start with N02A. A few other opioids are also relevant as analgesics, such as methadone and codeine. These drugs are not routinely included in the usual opioid use statistics, because they are not only indicted for analgesia (Jarlbaek, 2019). In this case, codeine combined with other analgesics was also excluded as these drugs may be used in other pathologies. This type of classification was used by Svendsen et al. (2011), Jones et al. (2020), Jarlbaek, (2019) and by Oliva et al. (2022) in their studies on opioid consumption. The subgroups of the ATC classification studied here are N02AA (natural opium alkaloids), N02AB (phenylpiperidine derivatives), N02AE (oripavine derivatives), N02AJ (opioids combined with other pain relievers, except codeine with combinations) and N02AX (other opioids).

This study used raw data from wholesalers supplying community pharmacies at the population level in the province of Las Palmas for the period 2016–2020. The data were provided by the Pharmaceutical Cooperatives of the Canary Islands (COFARCA) and the Spanish Pharmaceutical Cooperative (COFARES). The data collected cover the entire distribution of the drug in the evaluated area, since there are no other supply channels that could disturb the results obtained. Only data on opioids sold in pharmacies under prescription were included (AEMPS, 2020). The data provided were the number of packages sold of the different pharmaceutical preparations according to the ATC classification, the date of sale, the national code and the name of the pharmaceutical preparation (active pharmaceutical ingredient (API), dose, strength and units). The postcode (ZIP), which provides information on the municipality where the community pharmacy is located, but not its identity, was also included, thus maintaining its anonymity in accordance with current Spanish data protection legislation (Data Protection Law in Spain, 2018). The dataset contained the prescriptions corresponding to all residents of the province of Las Palmas who are registered in the municipal population register and have access to the Spanish national health service. The Spanish national healthcare system guarantees a universal healthcare system to all residents of Spain. For this reason, the data correspond to the medicines that are dispensed in pharmacies by the public health system, but we do not have data on hospitals.

The use of prescription opioids for each API was expressed in population doses per day, equivalent to the defined daily dose (DDD) per 1,000 inhabitants per day, where the number of DDDs was the total amount of the API consumed in a certain time period (in this case, day) divided by the DDD.

$$\frac{DDD\;x\;1000\;inhabitants}{day}=\frac{DDDs\;x\;1000}{populations\;x\;365\;days}$$ (1)

where DDDs represents the total number of DDDs,

$$DDDs=\frac{n\;x\;UD\;x\;N}{DDD}$$ (2)

where n is the number of packages dispensed, UD is unit dose in mg, N is the number of units per package, and DDD is the defined daily dose.

Data for Spain as a whole were provided by the Spanish Agency of Medicine and Medical Devices (Agencia Española de Medicamentos y Productos Sanitarios, AEMPS) as dispensation data under prescription in community pharmacies (AEMPS, 2020). Population data were downloaded from a publically accessible demographic database and are shown in Table 1 (National Statistics Institute, 2022). The database and dynamic tables of the Microsoft Excel^®^ program were used for the data processing.

**TABLE 1:** Demographic data and administrative distribution of the province of Las Palmas. The population density (inhabitants per km^2^), over-65-year-olds (%) population and median household income (€/year) corresponds to the 2020 years.

			Population			Population density	Over-65 years (%)	Income (€/year)
Island	2016	2017	2018	2019	2020	2020	2020	2020
Gran Canaria	845,195	843,158	846,717	851,231	855,521	548.4	16.97	11790
Lanzarote	145,084	147,023	149,183	152,289	155,812	184.2	12.57	11892
Fuerteventura	107,521	110,299	11,3275	116,886	119,732	72.1	11.11	10914
Province	1,097,800	1,100,480	1,109,175	1,120,406	1,131,065	278.2	13.07	11657

### 2.2 Statistical analysis

The following model based on covariance analysis (ANCOVA) with two nested fixed factors and one co-variable was used to analyze variation of each response during the 2016–2020 period:

$$y_{ij}=\beta_{0}+\left(\beta_{1+}\beta_{2i}x_{i}\right)*A_{i}+\beta_{3}B_{j,i}+\epsilon_{ijk}$$ (3)

where y_ij_ is the dependent variable (i.e., response) and x_i_ is the time (in years). The two factors used were: A_i_ is the effect of the *i*th island (i = 1 … 3) and B_j,i_ is the effect of the *i*th municipality within the *i*th island (j = 1.5 for i = 1; j = 1 .7 for i = 2; j = 1 … 21 for i = 3) and Є_ijk_ is the error term assuming it is independent with normal distribution with zero mean and constant variance (N ∼ (0, σ^2^)). The interaction “island x time” was included in order to analyze the variation of the drug’s prescription with time. In order to do this, the function **lm ()** from R-program was used. Tukey´s test was used in the comparison between two levels using the function **pairwise. t.test ()**. Marginal means were also computed at the different levels using the function **emmeans ()**, whereas the differences between each level was achieved through contrast analysis. All these functions are available in the free R-program (R Core Team, 2021).

Extension of this model to m factors is straightforward. In this context, the median household income was used to measure the socioeconomic level of each municipality and island, whereas the percentage of over 65 years old in population and population density were used as sociodemographic variables. All these data were downloaded from a publically accessible demographic database (National Statistics Institute in Spain and Instituto Canario de Estadística, Istac, 2022).

## 3 Results

### 3.1 Analysis by overall DDD per 1000 inhabitants per day

**FIGURE 1:**
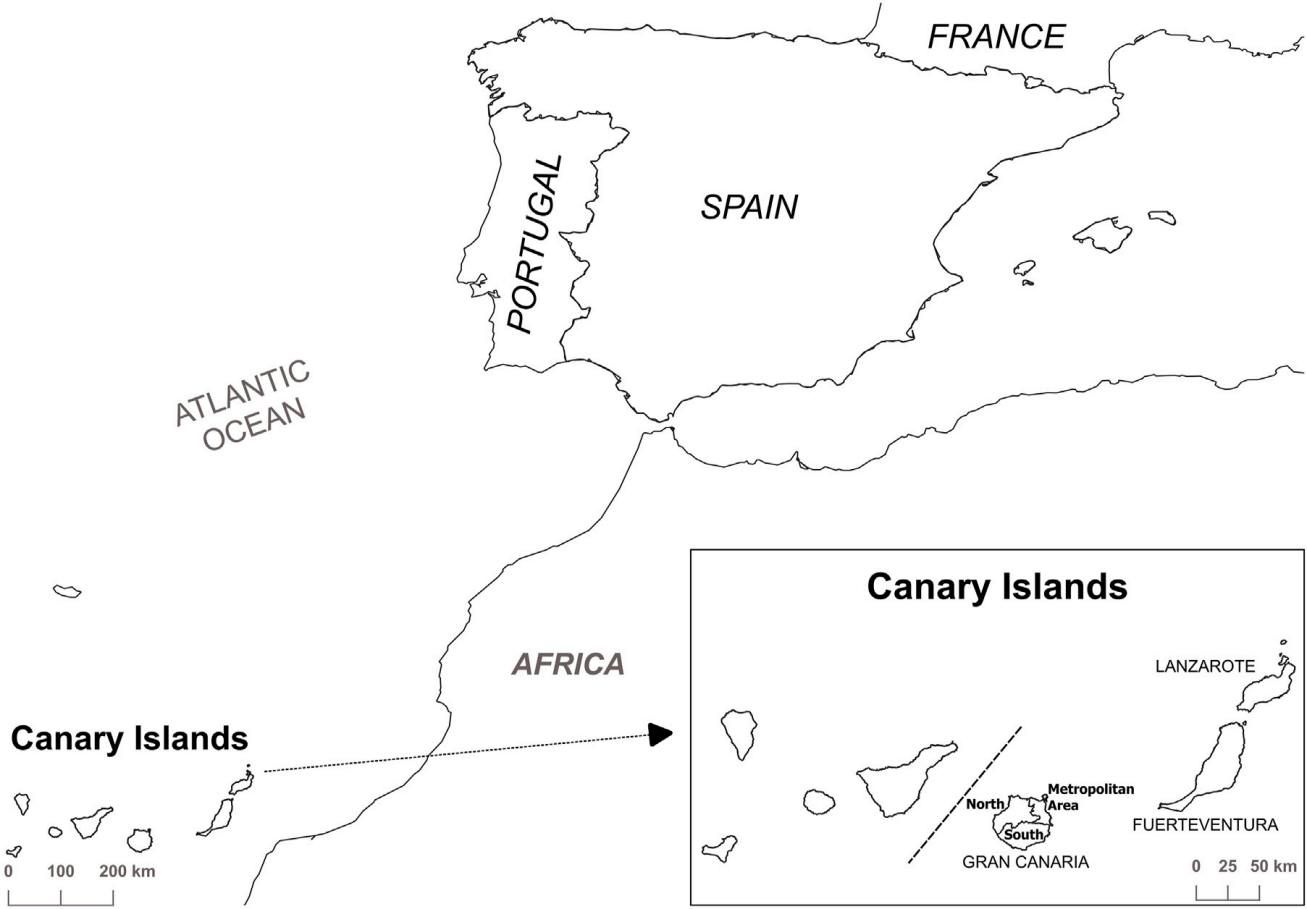
Map of the Canary Islands, Spain, with the three islands that make up the province of Las Palmas.

Figure 1 shows the map of the three islands, Gran Canaria, Lanzarote and Fuerteventura, that make up the province of Las Palmas, Canary Islands, Spain. Table 1 shows the population and demographic data at the provincial and island level. During the analyzed period, the population at the provincial level increased by 0.95%, with this increase being similar in Lanzarote and Fuerteventura (2.31% and 2.43%, respectively), whereas the increase in Gran Canaria was only 0.5%.

However, Gran Canaria is considered an urban area (Reig et al., 2016), with 44.6% of the population in the capital, which is home to the regional administration buildings, the reference hospitals and other essential services. Lanzarote and Fuerteventura are considered semi-urban, but compared with Lanzarote, Fuerteventura has the lowest population density (2.55 times lower) with proportions of elderly people of 11.11% and 12.57%, respectively. Additionally, the 41.5% of Lanzarote’s population lives in the capital (see Figure 1).

The overall DDD per 1000 inhabitants per day variation shows different trends in opioid use, for example, Gran Canaria presented the highest value, it was even higher than that those observed at a nationwide level, by approximately eight points, whereas Lanzarote and Fuerteventura had lower values (Table 2). However, the trends in both islands were different; Fuerteventura had an increasing tendency, whereas Lanzarote show a stable trends during this period, although the consumption in 2020 was negative, a decrease of 16.5% was observed in comparison to the year 2019, whereas the other geographical areas consumption increased in the in the range 2.37% at the provincial level and 7.90% in Fuerteventura compared to 9.20% found for Spain as a whole (see Figure 1).

**TABLE 2:** Variation of the overall DDD per 1000 inhabitants per day and percentage of strong opioids, expressed as a percentage of overall DDD per 1000 inhabitants per day at different levels (province and island) during the period 2016–2020.

Overall DDD per 1000 inhabitants per day				
Year	Province	Gran Canaria	Lanzarote	Fuerteventura
2016	19.62	22.66	9.73	9.83
2017	21.38	24.48	10.68	11.94
2018	22.00	25.20	10.82	12.84
2019	22.82	26.29	10.90	13.04
2020	23.36	27.26	9.10	14.07
% Strong opioids				
2016	56.66	55.93	54.05	57.91
2017	53.94	54.02	51.46	59.56
2018	52.31	51.75	47.04	58.48
2019	51.62	51.46	43.74	56.13
2020	51.98	49.90	59.71	59.41

Firstly, the prescription rate would be expected to increase with the rise in the number of aged people (expressed as a percentage of over sixty-five-year olds in the population). The results show that there is no significant correlation between the variation of overall DDD per 1000 inhabitants per day and this variable in all the municipalities for each island (r < 0.05). A similar result was obtained for the other two variables: population density and median household income, although Lanzarote shows a positive correlation between overall DDD per 1000 inhabitants per day and population density (r = 0.593). This result is fairly misleading since the population density in Arrecife is fifteen times higher than the mean average of the rest of the island.

The second step was to analyze the variability in overall DDD per 1000 inhabitants per day between-islands and municipalities during the study period, using an ANCOVA following the model proposed in Material and Methods (Table 3). Firstly, the coefficient of adjusted determination (*R* ^2^) was 0.947 and thus, the model proposed is suitable for the data interpretation. In addition, demographic and socio-economic characteristics were included in the model as factors such as income (€/year), percentage of over sixty-five-year olds in the population, population density and interaction term. This new model allowed *R* ^2^ to increase up to 0.9585, but these new terms were not statistically significant and the simpler model was used. The null hypothesis for the island factor was rejected (*p* < 0.05), and it was also rejected for the interaction term “island x year”. This fact supposes that the overall DDD per 1000 inhabitants per day varied between islands during the study period. Tukey’s test confirmed this issue (*p* < 0.01). In addition, the overall DDD per 1000 inhabitants per day variation rate varied between-islands as indicated in the term “island x year” (Table 4). In this case, Gran Canaria and Fuerteventura had the same variation rate (1.004 DDD per 1000 inhabitants per day and year), whereas the island of Lanzarote showed a negative variation rate (−0.193 DDD per 1000 inhabitants per day and year). However, Lanzarote and Fuerteventura presented the same dispensation level (i.e., intercept), whereas Gran Canaria had a higher level (18.245 vs. 12.697 DDD per 1000 inhabitants per day). Furthermore, the data from Table 3 shows that there are differences between municipalities from different islands (this is logical) but also within the same island. In order to confirm this result, a second analysis was performed to determine the possible differences between municipalities for each island as well as their evolution during this period (Table 5), and the marginal means were calculated to do this. The results shown from Table 5 demonstrate that all pairwise differences are statistically significant (*p* < 0.05).

**TABLE 3:** Results of the ANCOVA according to the two nested factor model for the overall DDD per 1000 inhabitants per day response (*) Significance for *p* < 0.05.

Source of variation	Df	Sum of squares	Mean square	F	Pr<|t|
year	1	161.2	161.2	36.5	<2·10^−6^ *
island	2	7804	3902	882	<2·10^−6^ *
year x island	2	70.3	35.2	7.95	5.55 · 10^−4^ *
island x municipalities	30	5121	170.7	38.6	<2·10^−6^ *
residuals	129	570.2	4.4		

**TABLE 4:** Results of ANCOVA for the factors island and island x year, respectively according to the nested model for the overall DDD per 1000 inhabitants per day and percentage of strong opioid (SO) (expressed as a percentage of overall DDD per 1000 inhabitants per day) 1) Data refer to Fuerteventura (reference level) (*) Significance for *p* < 0.05.

Overall DDD per 1000 inhabitants per day				
Coefficients	Estimate	2.5%	97,5%	Pr (>|t|)
Intercept (1)	12.697	10.133	15.261	<0.01 *
Year (1)	1.004	0.416	1.592	9.68 · 10^−4^ *
Gran Canaria	5.548	2.265	8.831	<0.01 *
Lanzarote	−2.559	−0.606	0.943	0.151
year: Gran Canaria	−0.0820	−0.736	0.573	0.805
year: Lanzarote	−1.193	−1.963	−0.422	2.65 · 10^−3^ *
% Strong opioid				
Intercept (1)	56.41	50.86	61.96	<2·10^−6^ *
Gran Canaria	−38.80	−45.9	−31.69	<2·10^−6^ *
Lanzarote	−28.08	−35.67	−20.51	<2·10^−6^ *
Year (1)	−0.0579	−1.331	1.215	0.928
year: Gran Canaria	0.4070	−1.010	1.823	0.571
year: Lanzarote	1.667	−1.118	3.333	0.0521

**TABLE 5:** Estimated marginal means and 95% confidence intervals for overall DDD per 1000 inhabitants per day (DDD/1000inh/day) and percentage of strong opioid expressed as a percentage of overall DDD per 1000 inhabitants per day (%SO) for the different islands. SE: Standard error with 129 degrees of freedom.

Island	DDD/1000 inh/day	SE	95% CI	%SO	SE	95% CI
Fuerteventura	13.0	0.420	12.2, 13.8	61.2	0.910	59.4, 63.0
Gran Canaria	24.2	0.205	22.8, 24.6	22.4	0.444	21.5, 23.3
Lanzarote	8.22	0.355	7.52, 8.92	30.1	0.769	28.6, 31.6

Table 6 shows the estimated marginal means for different municipalities within the same island. The contrast analysis identified differences between municipalities within the same island (*p* < 0.05).

**TABLE 6:** Estimated marginal means and 95% confidence intervals for overall DDD per 1000 inhabitants per day (DDD/1000inh/day) and percentage of strong opioid for the different municipalities on each island. The residual standard error was 0.94 for DID response and 2.03 for percentage of strong opioid response on 129 degrees of freedom.

Municipalities in Fuerteventura		
Municipality	DDD/1000 inh/day	% Strong opioids
ANTIGUA	14.69 [12.83, 16.55]	61.1 [57.03, 65.1]
LA OLIVA	8.36 [6.50, 10.22]	61.9 [57.84, 65.9]
PÁJARA	13.87 [12.01, 15.73]	68.3 [64.32, 72.4]
PUERTO DEL ROSARIO	12.42 [10.56, 14.28]	58.3 [54.25, 62.3]
TUINEJE	15.71 [13.85, 17.57]	56.2 [52.21, 60.3]
Municipalities in Gran Canaria		
AGAETE	21.01 [19.15, 22.87]	18.7 [14.64, 22.7]
AGÜÍMES	19.98 [18.12, 21.84]	23.7 [19.69, 27.7]
ARTENARA	8.40 [6.54, 10.26]	33.1 [29.11, 37.2]
ARUCAS	27.34 [25.48, 29.20]	25.3 [21.26, 29.3]
FIRGAS	18.95 [17.09, 20.81]	24.6 [20.54, 28.6]
GÁLDAR	27.08 [25.22, 28.94]	17.3 [13.26, 21.3]
INGENIO	29.27 [27.41, 31.13]	24.2 [20.17, 28.2]
LA ALDEA DE SAN NICOLÁS	29.54 [27.68, 31.40]	23.1 [19.08, 27.1]
LAS PALMAS DE GRAN CANARIA	24.54 [22.68, 26.40]	22.8 [18.77, 26.8]
MOGÁN	21.18 [19.32, 23.04]	23.9 [19.83, 27.9]
MOYA	39.68 [37.82, 41.54]	22.1 [18.10, 26.2]
SAN BARTOLOMÉ DE TIRAJANA	20.32 [18.46, 22.18]	23.4 [19.37, 27.4]
SANTA BRÍGIDA	18.91 [17.05, 20.77]	20.7 [16.67, 24.7]
SANTA LUCÍA DE TIRAJANA	26.17 [24.31, 28.03]	26.0 [21.93, 30.0]
SANTA MARÍA DE GUÍA	25.58 [23.72, 27.44]	22.1 [18.10, 26.2]
TEJEDA	11.80 [9.94, 13.66]	11.0 [7.02, 15.1]
TELDE	29.36 [27.50, 31.22]	22.8 [18.76, 26.8]
TEROR	27.81 [25.95, 29.67]	21.8 [17.73, 25.8]
VALLESECO	26.70 [24.84, 28.56]	30.0 [26.01, 34.1]
VALSEQUILLO	23.84 [21.98, 25.70]	17.7 [13.63, 21.7]
VEGA DE SAN MATEO	30.80 [28.94, 32.66]	16.2 [12.17, 20.2]
Municipalities in Lanzarote		
ARRECIFE	14.83 [12.97, 16.69]	25.2 [21.2, 29.3]
HARÍA	7.97 [6.11, 9.83]	23.8 [19.74, 27.8]
SAN BARTOLOME	5.32 [3.46, 7.18]	28.3 [24.23, 32.3]
TEGUISE	4.76 [2.90, 6.62]	32.3 [28.3, 36.4]
TÍAS	9.31 [7.45, 11.17]	23.8 [19.82, 27.9]
TINAJO	5.77 [3.91, 7.63]	44.2 [40.14, 48.2]
YAIZA	9.57 [7.71, 11.43]	33.2 [29.13, 37.2]

**FIGURE 2:**
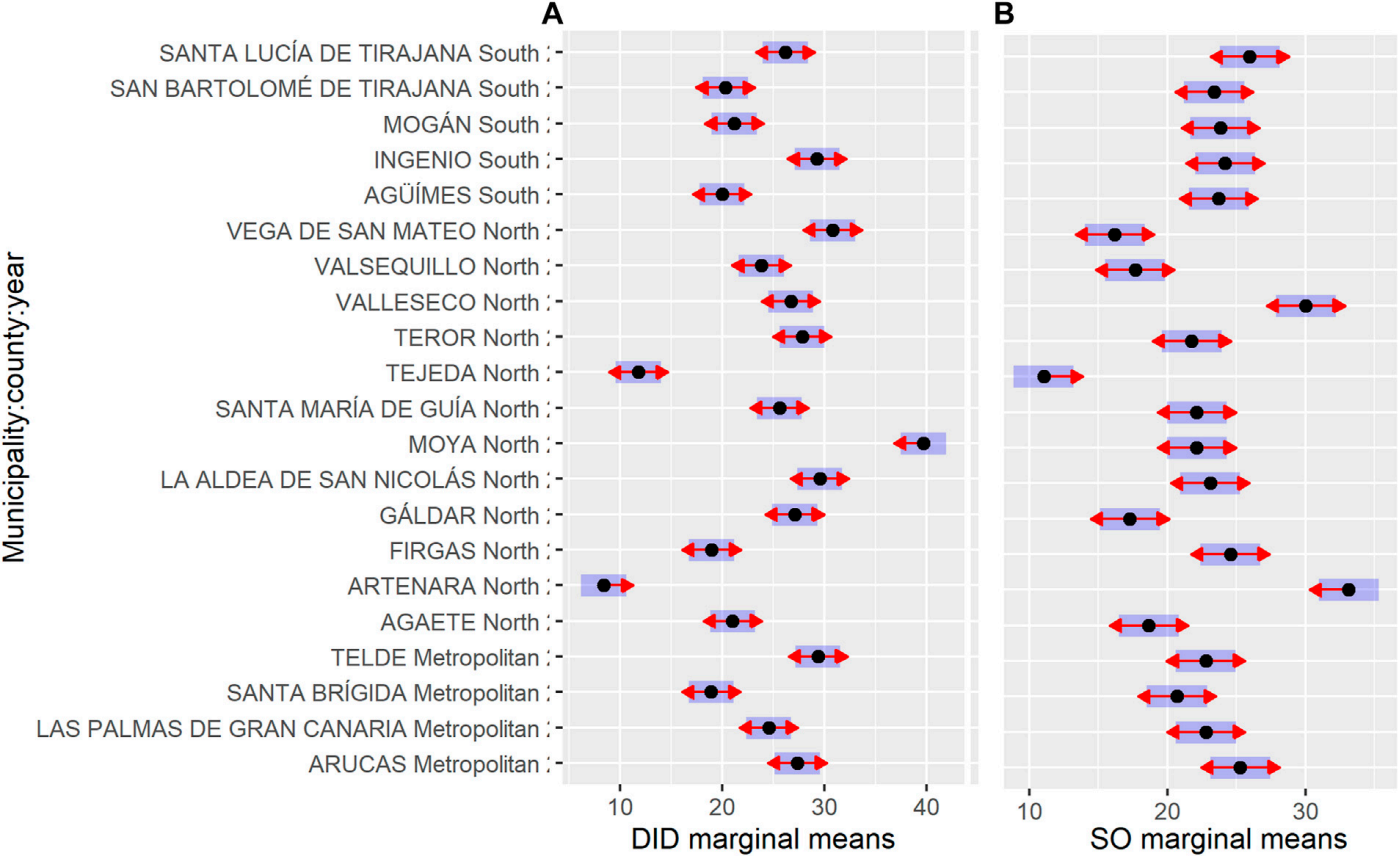
Estimated marginal means for the different municipalities within each district on the island of Gran Canaria for DDD per 1000 inhabitants per day **(A)** and percentage of strong opioid responses **(B)**.

From an administrative point of view, the island of Gran Canaria has twenty-one municipalities and, the contrast analysis shows more than 100 pairwise entries and, therefore, the data interpretation is too complex. In such a situation, the analysis of the data set has been in terms of district (see Figure 1). Gran Canaria is divided into three districts: metropolitan district (4 municipalities), northern district (12 municipalities) and southern district (5 municipalities). Table 7 shows the results obtained in the contrast analysis, indicating that there are no differences between districts (*p* > 0.05). Figure 2 shows the estimated marginal means for the different municipalities within each district of Gran Canaria, indicating there are differences between municipalities within the same district.

**TABLE 7:** Estimated marginal means and 95% confidence intervals for overall DDD per 1000 inhabitants per day (DDD/1000inh/day) and percentage of strong opioid responses (% SO) for different districts in Gran Canaria. SE: Standard error with 129 degrees of freedom.

District	DDD/1000 inh/day	SE	95% CI	% SO	SE	95% CI
Metropolitan	25.0	0.553	23.9, 26.1	22.9	0.546	21.8, 24.0
Northern	24.3	0.319	23.6, 24.9	21.5	0.315	20.8, 22.1
Southern	23.4	0.495	22.4, 24.4	24.2	0.481	23.3, 25.2

### 3.2 Data analysis on strong opioids

Opioids are classified in two groups: weak (tramadol in monotherapy and combinations) and strong opioids (the remaining ones). In this case, codeine combined with other analgesics was excluded as these drugs may be used in other pathologies. At the island level, the data analysis based on this classification shows that the strong opioids, expressed as a percentage of overall DDD per 1000 inhabitants per day (SO) are the most consumed in Fuerteventura, whereas on Gran Canaria and in Spain as a whole, SO account for less than 30% (on average) and with a decreasing tendency (Table 2). Lanzarote shows the same pattern but a sharp increase was observed in 2020, although the proportion of elderly people rate is the lowest of the three islands.

The results of ANCOVA for the SO, show that the proposed model is also suitable for the data interpretation as the coefficient of adjusted determination (*R* ^2^) is 0.9093. The null hypothesis for the island factor was rejected (*p* < 0.05), whereas it was accepted for the interaction term “island x year” (data not shown). This means that the SO varied between islands during the study period (Tukey’s test confirmed this issue *p* < 0.01). Thus, the dispensation level (i.e., intercept) was different, 56.41% in Fuerteventura *versus* 17.61% in Gran Canaria, although this has remained fairly stable in all three islands over the last 5 years. In this regard, the estimated value for the consumption rate of SO is zero for all three islands since the 95% confidence interval included the zero value (Table 4), although this data should be confirmed for Lanzarote since the probability level obtained was slightly higher than 5% (*p* = 0.0521) but with an increasing tendency (+1.610 SO per year).

The analysis of estimated marginal means shows that there are differences between municipalities within the same island (Table 6). In the case of Gran Canaria, the contrast analysis was performed at the district level (Figure 1). Table 7 shows the results obtained, indicating that all the differences were not significant (*p* > 0.05), except the northern-southern contrast (*p* < 0.001). Besides which, the data analysis by municipalities within each district shows that there are no differences between the municipalities in southern district, whereas the difference between Arucas and Santa Brigida municipalities was significant in the case of the metropolitan district (Figure 2). The northern district shows differences between its municipalities, although all of them are considered rural areas (Figure 2).

### 3.3 Pharmaceutical preparation prescription

The combination of tramadol and paracetamol in tablet form was the most commonly prescribed formulation in all three islands. It was produced and packaged with a low dose and low number of units per package (37.5 mg/325 mg; 20 units) and in 2016 the presentation was changed keeping the same dose but with a higher number of units per package (60 units). In the case of the first presentation, the values (expressed as percentage of units sold per 1000 inhabitants and year) varied between 77.14% for Gran Canaria and 86.12% for Lanzarote, whereas in the second case, the mean value was 12.12% with a standard deviation of 1.17%. However, a trend in change was observed in 2020, with an increase in the consumption of the latter presentation, 2.4-fold (on average), whereas the high-dose and high-number of units per package presentations (75 mg/650 mg; 60 units) saw its consumption double in the same period, although the dispensation level was higher in Gran Canaria, 16.39% *versus* 2.30% in Fuerteventura. This pattern was mainly observed during the year 2020, although the preliminary data analysis for the year 2021 seems to confirm this finding (data not shown).

Tramadol indicated in monotherapy is prescribed as long-acting and low-dose (50 mg/20 units) tablets, although its demand varied between islands. For example, a slight increase was observed in Lanzarote during 2020 in comparison to 2016 (51.75% vs. 45.57%), whereas, the increase was of the same order of magnitude in Fuerteventura but its consumption was the half (20.64% vs. 26.59%), since the presentation with the same doses but high-units per package (60 units) was the most frequent option but with a negative tendency in this period (26.42% vs. 34.27%). Gran Canaria presented the same trends as Fuerteventura, although the consumption rates were different, a mean of 29.78% *versus* 15.37% for low dose and low-units per package. However, it should be mentioned that the presentations of 100 mg and 60 units per package had a consumption rate of 14.01% in 2016 but with an increasing tendency in 2020, reaching levels of 18.30%. It is noteworthy that the oral solution pharmaceutical form had a demand close to 3% of the total in 2020, which is prescribed for older people because of its easy administration.

However, differences in prescribing SO use between islands were observed, especially between Lanzarote and Fuerteventura. For example, in Fuerteventura, the prescription buprenorphine use is characterized by low-dose and long-acting preparations (transdermal patches; 35 µg; 5 units), accounting for 33.25% in 2016, but this tendency decreased to 26.73% in 2020. A similar situation was observed for fentanyl presentations (25µg/5 patches), accounting for 34.90% in 2016 and 28.05% in 2020. In Lanzarote, the consumption rate of buprenorphine was fairly stable at 41% on average in the 2016–2020 period, followed by fentanyl presentations (25 µg/5 patches) which accounted for 22.45% on average. In Gran Canaria, the prescription of buprenorphine patches (35 µg/5 units) with 28.15% of the total was the most consumed followed by fentanyl patches at low-dose (21.73% on average), both consumptions remained stable during the analyzed period.

The prescription patterns at the municipal level were similar to those observed at the island level, although with small variations in the dispensation level and/or in the ranking between the more consumed formulations and presentations.

## 4 Discussion

In the investigation of prescription opioid use, differences in measurement tools and the selection of opioids should be taken into consideration if the aims are to compare and report results on opioid use between geographical areas (countries, regions, islands, municipalities, etc.) and changes over time in order to know the trends in consumption.

Quantification of opioid consumption and dosage opioid doses can be quantified in several ways, most commonly in defined daily doses (DDDs) or oral morphine equivalent doses (MME) (García del Pozo et al., 2008; Svendsen et al., 2011; Hurtado et al., 2020; Böckerman et al., 2021a; Perry et al., 2022). Most national prescription databases report opioid consumption in DDDs, a unit recommended by the WHO for drug consumption studies. In a such situation, the Spanish Agency for Medicines and Health Products (AEMPS, 2020) uses DDDs per 1000 inhabitants per day for the quantification of opioid use. Several studies conducted in European countries and some local studies in Spain (at regional level) have also used this unit of measurement (García del Pozo et al., 2008; Hurtado et al., 2020; Böckerman et al., 2021b; Serra-Pujadas et al., 2021; Oliva et al., 2022). This allows the comparison of opioid use between different geographical areas. In the present study, the opioids indicated for analgesia, excluding methadone and codeine combined with other analgesics, were analyzed (Oliva et al., 2022). In the present study, and for comparative purposes, the data of the nationwide report by AEMPS were re-analyzed to take this fact into account (Oliva et al., 2022). The recalculated DDD per 1000 inhabitants per day varied from 14.75 in 2016 to 18.59 in 2020, values that are below the overall DDD per 1000 inhabitants per day at the province level and on the island of Gran Canaria, whereas DDD per 1000 inhabitants per day in Lanzarote was only the half of the nationwide value. However, the overall DDD per 1000 inhabitants per day and year variation rate in Spain was very similar to those found in Gran Canaria and Fuerteventura, although the levels of dispensation were different. Lanzarote is completely different in all cases, where the opioid consumption rate remained stable during the studied period, but with a negative tendency; the data for the year 2021 seems to confirm this (data not shown). The dispensation level of SO varied between islands, although these values remained stable because a consumption rate equal to zero was observed during the study period (Table 4). Lanzarote had very similar values to those observed in Spain as a whole (30.0% vs. 31.24%).

Opioid prescription increased in nearly all European countries from 2006 to 2016, although with marked differences between countries (Häuser et al., 2021). Spain as a whole had one of the lowest rates of opioid consumption in comparison with other European countries (Hurtado et al., 2020) According to the AEMPS report, opioid consumption in Spain nearly doubled in the decade 2010–2020 (10.03 DDD per 1000 inhabitants per day in 2010 to 19.91 DDD per 1000 inhabitants per day in 2020.i.e., ∆ = 98.5%), although in the last 5 years (2016–2020), the increase was lower, 25.1% and with a positive tendency. Hamunen et al. (2009) conducted a study of opioid consumption in three Scandinavian countries between 2006–2014 in terms of DDD per 1000 inhabitants per day, which showed that Denmark presented DDD per 1000 inhabitants per day values close to those observed in Spain as a whole (≈20 DDD per 1000 inhabitants per day), whereas Norway had the lowest consumption (≈17 DDD per 1000 inhabitants per day). Chenaf et al. (2019) analyzed the trends in prescription opioids use in France between 2004–2017 and found that opioid prescription more than doubled during this period, which was associated with a nontrivial increase in opioid-related morbidity-mortality. However, in the last 10 years, the pattern in the volume of opioid prescription has been stable in most European countries (Hurtado et al., 2020).

The model proposed to evaluate the variability between islands, municipalities or districts for the two analyzed responses was appropriate since the *R* ^2^ value was greater than 90%. The estimated marginal means analysis shows that there are differences between islands, municipalities and districts in the case of the island of Gran Canaria. At the island level, these differences in prescription patterns could be related with the geographic area itself and its demographic characteristics and its economy which is closely linked to tourism and/or the primary sector.

In such a situation, the municipality of Arrecife, capital of the island of Lanzarote, is considered an urban touristic have the highest DDD per 1000 inhabitants per day. The municipalities of Tias and Yaiza are considered as semi-urban areas, with agriculture and tourism being their main economic activities, whereas the municipalities of Tinajo, San Bartolomé and Teguise, considered as mainly rural areas, have the lowest DDD per 1000 inhabitants per day, with less population density and a greater proportion of elderly people in comparison to the semi-urban areas. In addition, the tourist activity is small in comparison with other zones.

However, the municipality of Tinajo, the most rural in Lanzarote, is markedly different, with the level of SO prescription being 1.5 times higher than those observed at the island level (44.1% vs. 30.1%), followed by the municipalities of Yaiza, Teguise and San Bartolomé, which have SO above the island average (32.7% vs. 25.0%), while the rest of municipalities, including Arrecife, have SO below 25% (on average), although the differences between them are significant.

In Fuerteventura, the municipality of La Oliva, located in the north of the island, is a semi-urban area whose economic activity is linked to tourism and had the lowest value of DDD per 1000 inhabitants per day compared to the rest of the municipalities. These other municipalities are rural areas, whose economic activity is in the primary sector and to lesser extent tourism, except Puerto del Rosario, the island’s capital, which is also considered a semi-urban area, where the tourism and services sector (administration) is the main economic activity, although its DDD per 1000 inhabitants per day is similar to those observed in rural municipalities. However, the municipalities of Antigua, Pajara and Tuineje, with the smallest population density (50 inhabitants/km^2^), had DDD per 1000 inhabitants per day values above the annual one at the island level, with the largest proportion of elderly people, 16% *versus* 11.1%. A similar situation was observed for SO, where there is a statistically significant difference between Pajara compared to both Puerto del Rosario and Tuineje.

A detailed analysis per district was carried out in Gran Canaria. In the case of the metropolitan district, all differences were significative for the Santa Brigida municipality (*p* < 0.01), whereas Las Palmas de Gran Canaria, the capital of the island, also differs from the Telde municipality (*p* < 0.0148). These two municipalities are situated in the surrounding area of the capital and are within easy reach of the capital. Telde has a higher proportion of residents who work in manual jobs, industry and to a lesser extent in the agricultural sector, these people usually suffer work-place related physical injury and chronic pain. This fact could explain the greater DDD per 1000 inhabitants per day rates observed since Telde has the lowest proportion of elderly people, 14.56% compared to Santa Brigida or the capital above 18%. On the other hand, the Santa Brigida municipality is a residential zone with easy access to the capital (<15 min), where many residents live in the town, but they travel daily to capital to work in the services and administration sector. In addition, this municipality has the highest rates of income in the Canary Islands, €16,634/year compared to €12,008/year (average annual income) for the remaining metropolitan district municipalities. However, the DDD per 1000 inhabitants per day rate is the lowest in the metropolitan area, although the proportion of elderly people is slightly higher than the island average (18.9% vs. 17.0%).

Although all municipalities in the northern district are considered rural areas, there are differences between them. In this context, it is possible to distinguish between two types of municipalities in terms of accessibility to urban areas. At this point, it seems that there is an imaginary line that divides the north district is two-halves: on the left of the line, we find the more ruralized municipalities with lower population densities, population aging, geographical isolation, regressive economic conditions due to excessive dependence on a low-productive agricultural sector and longer access times to the urban center (>60 min). The municipalities of Tejeda and Artenara, characterized by the lowest population densities (<20 inhabitants/km^2^) and a high proportion of elderly people (>26%), are good example of this situation. However, their DDD per 1000 inhabitants per day values are the lowest.

Both municipalities have extreme SO values, 11.0% vs. 33.1%, respectively (Figure 2). In this context, the number of units sold was markedly different, with Artenara accounting for 37.1% of the sales corresponding to the SO, while Tejeda accounts for 9.21%, all data corresponding to the year 2020, although both municipalities show different trends in the period analyzed, with an increasing positive trend in the case of Tejeda, while the trend remains stable in Artenara.

On the right-hand side of the line have the municipalities of Teror, Moya, or Firgas as example, with the highest population densities in the range of between 250 and 500 inhabitants/km^2^. However, this data could be misleading as these three municipalities are the smallest in terms of surface area. In addition, they all have an elderly population of more than 17%, with a low socio-economic status, but with easy access to urban centers (<15 min) as they are located on the border of the metropolitan district. However, the values of DDD per 1000 inhabitants per day vary considerably, from 18.95 in Firgas to 39.68 for Moya.

However, the SO in these three municipalities was similar to the district average, although the greatest variability was observed in Moya. The number of units sold was very similar between these municipalities, with an average of 19%. However, Moya had a value of 25.5% and the proportion of elderly people was 20.5%.

In the southern district, all differences found in the municipality of Ingenio were significant, except for contrasting with the municipality of Santa Lucia (*p* = 0.285). Ingenio is a rural area, where agriculture is the main economic activity, although there is a growing services sector. However, it has the highest value of DDD per 1000 inhabitants per day value (29.27 DDD per 1000 inhabitants per day), although the proportion of elderly people is low, 13.14%, whereas the municipalities of Aguimes, Mogan or San Bartolome de Tirajana have similar values of DDD per 1000 inhabitants per day (20 on average), but with a proportion of elderly people of over 15%. These three municipalities are urban areas with tourism is the most important economic activity. A similar situation was observed in Santa Lucía, which differs from these three municipalities in that it is not only the most urban in the southern district, with the highest population density (1,212 inhabitants/km^2^), but also has the lowest percentage of elderly people (10.44%) and an economy based on tourism. However, despite having very different demographic and socio-economic characteristics, the differences between them do not reach significance for the SO. Mordecai et al. (2018) demonstrated that prescription opioid use rates were higher in economically deprived areas; other studies report that elderly women who live alone or in a rural area have the highest rates of opioid prescription (Keyes et al., 2014; Serdarevic et al., 2017). Serra-Pujadas et al. (2021) showed that opioid use (expressed as DDDs per 1000 inhabitants per day) in Catalonia, Spain, was higher in the fourth major urban region and in the surrounding area of the Barcelona metropolitan area. The findings showed that DDD per 1000 inhabitants per day were 10 times higher in the areas with the highest use than in those with the lowest. The socio-economic and demographic characteristics of the areas could explain these differences.

In the case studied here, factors such as the proportion of older people, socioeconomic status, population density or access to healthcare services help to explain the opioid prescription rates and prescription pattern in all the islands and their municipalities. Firstly, the prescription rate should increase in line with the rate of the rise in the population’s age. The results show that there is no significant correlation between the prescription opioid use rate, expressed as DDD per 1000 inhabitants per day and SO, and the population-aging rate at the municipal level. A similar result was found for income and population density variables (data no shown).

With regard to the main drugs contributing to the upward trend, buprenorphine and fentanyl are the greatest contributors among the strong opioids, while in the case of weak opioids tramadol alone or in combination with acetaminophen are the greatest contributors, although with differences between islands. The strong opioids accounted for 63.45% of the total units sold in 2020 in Lanzarote *versus* 49.88% in Gran Canaria. All of the above mentioned are prescribed for the treatment of pain with precise indications. Buprenorphine is indicated for treatment of moderate to severe cancer pain and severe pain that does not respond to non-opioid analgesics as well as in patients with pain who have an active opioid use disorder (Dowell et al., 2022), whereas fentanyl is indicated for cancer patients with breakthrough pain, although its use has increased in non-cancer patients (González-Bermejo et al., 2021). This increase in prescription in Spain as well in other European countries could be related to the ease of administrating transdermal formulations (Bosetti et al., 2019; González-Bermejo et al., 2021).

As for the weak opioids, a change in prescription pattern was observed for tramadol in combination with acetaminophen, especially in Gran Canaria where an increase of total units sold with high-doses (75/650 mg) and high-units per package (60 units) was found during the study period, especially in year 2020. A possible explanation could be that physicians prescribed the high unit per package presentations to avoid unnecessary patient visits due to COVID-19 restrictions.

The dispensation level was two times higher in Lanzarote than in Fuerteventura (48.66% vs. 23.61%) for low-dose and low-unit per package presentations. All these formulations are indicated for the treatment of moderate to severe intensity pain, especially in older-people since it is prescribed in preference to NSAID drugs because of concerns over complications. In addition, the use of newer additions to the therapeutic arsenal, such as tapentadol or oxycodone plus naloxone were found to have very low consumption rates, whereas in Spain as a whole, the data seems to be suggesting an increase (AEMPS, 2020).

Hider-Mlynarz et al. (2018) studied the consumption trends of three groups of analgesics between 2006 and 2015 in France, and compared this pattern of use with six European countries in 2015. France ranked third in weak opioid consumption whereas the use of strong opioids in France was among the lowest. The UK had the highest consumption of weak and strong opioids among the six European countries analyzed. The difference in analgesic preferences observed between European countries may reflect the role of national guidelines, prescription policies and the marketing strategies of pharmaceutical companies, which can differ between these countries, but it can also be explained by cultural backgrounds and local traditions in managing chronic pain.

## 5 Conclusion

The results of the present study should be considered with the following limitations. Firstly, the information provided by the wholesalers who supply the community pharmacies is valuable, although limited since patients’ demographic data (age and gender) or medical data (pathology or duration of treatment) were not provided. However, with these data it was possible to reliably analyze the geographical distribution of prescription opioid use.

Secondly, the overall picture of prescription opioid use was different for each island and municipalities, even between districts as was the case in Gran Canaria. Two observations can be made at the municipality level: the most ruralized zones are located in the interior areas and far away from urban centers (longer access time). In these zones, most municipalities have lower population densities, higher rates of old age, low socio-economic status, geographical isolation with fewer public services. The urban areas are located in coastal areas and the capitals of each island are characterized by the existence of large population concentrations, their income levels are above the island average and they enjoy greater access to public services.

Thirdly, the datasets used in this study included dispensing data (from community pharmacies under prescription) and excluded hospital prescriptions, with the risk of underestimating true opioid use. However, these would mainly concern acute care, managed by primary care physicians and would therefore be captured in our database at some point in time. A similar situation was described by Xie et al. (2022) in Catalonia, Spain. In addition, prescription data are essentially an ‘intention to treat’ and may therefore overestimate opioid use. At the municipal level, the annual variation in prescription could be overestimated, although the bias is difficult to quantify since the prescription is associated to pharmacy postal code (ZIP) where the patient lives but they could get their medication in a pharmacy in another municipality. In a such situation, the small municipalities (<2,000 inhabitants) with a single community pharmacy in the province of Las Palmas have levels of variation in sales around 10%–20%. This finding was also observed on the islands of El Hierro and La Gomera (≈10–20%), the two smallest islands in the Canary archipelago (Oliva et al., 2022). On the other hand, in municipalities with two or more community pharmacies, the pattern was the same, although in some ZIPs, the level of variation was less than 10%, but it never exceeded 20%. For this, DDD per 1000 inhabitants per day data at island and province level are more accurate since they included all population who live in that zone.

The findings here are, in general, consistent with those obtained in other European countries, where a general pattern of growth in opioid consumption has been observed in the two last decades. However, there are some notable variations in the relative levels of consumption between different geographical areas and some distinct, context-specific patterns of use for some drugs. Prescription of strong opioids was higher than 50% (on average) in all the studied islands *versus* 31.7% in Spain as a whole. However, there are differences in prescription patterns of weak and strong opioids, as well as in the doses and units per package according to each island. In this respect, Lanzarote and Fuerteventura are characterized by having specialist doctors located in the capital (i.e., in only one place) who initiate the prescription according to an indication and dosage form. The authors believe that this factor plays an important role in determining opioid prescription rates. This type of pattern was also observed in the two smallest islands of the Canary Islands (Oliva et al., 2022). However, Gran Canaria has two reference hospitals located in the capital where the patients from all over the island are referred depending on where their first residence is and not on their illness. In the authors’ opinion, the opioid prescribing practices mentioned above as well as the social and cultural traditions could be determinants. However, other factors such urbanicity level, population age, socioeconomic status do not help to explain the differences in prescription opioid use across rural and urban areas.

Our findings call for stronger action to promote best practices in prescription opioids use and to reduce socio-economic and demographic variation between geographical areas. The model proposed by this study could be used for the routine monitoring of the prescription of opioids in order to promote interventions able to reduce the consumption in the islands, especially in Gran Canaria, and is certainly not intended to criticize legitimate medical uses.

## Data Availability

The raw data supporting the conclusion of this article will be made available by the authors, without undue reservation.
